# Regression of late gadolinium enhancement in a case of eosinophilic granulomatous polyangiitis with cardiac involvement

**DOI:** 10.1093/ehjci/jeaf163

**Published:** 2025-05-26

**Authors:** Pok-Tin Tang, Jonathan Raby, Jessica Gunn, Andrew J M Lewis

**Affiliations:** Oxford Centre for Clinical Magnetic Resonance Research, Division of Cardiovascular Medicine, Radcliffe Department of Medicine, University of Oxford, Headley Way, Oxford OX3 9DU, UK; Department of Cardiology, John Radcliffe Hospital, Headley Way, Oxford OX3 9DU, UK; Oxford Centre for Clinical Magnetic Resonance Research, Division of Cardiovascular Medicine, Radcliffe Department of Medicine, University of Oxford, Headley Way, Oxford OX3 9DU, UK; Department of Cardiology, John Radcliffe Hospital, Headley Way, Oxford OX3 9DU, UK; Department of Rheumatology, Gloucestershire Royal Hospital, Great Western Road, Gloucester GL1 3NN, UK; Oxford Centre for Clinical Magnetic Resonance Research, Division of Cardiovascular Medicine, Radcliffe Department of Medicine, University of Oxford, Headley Way, Oxford OX3 9DU, UK; Department of Cardiology, John Radcliffe Hospital, Headley Way, Oxford OX3 9DU, UK

**Figure:**
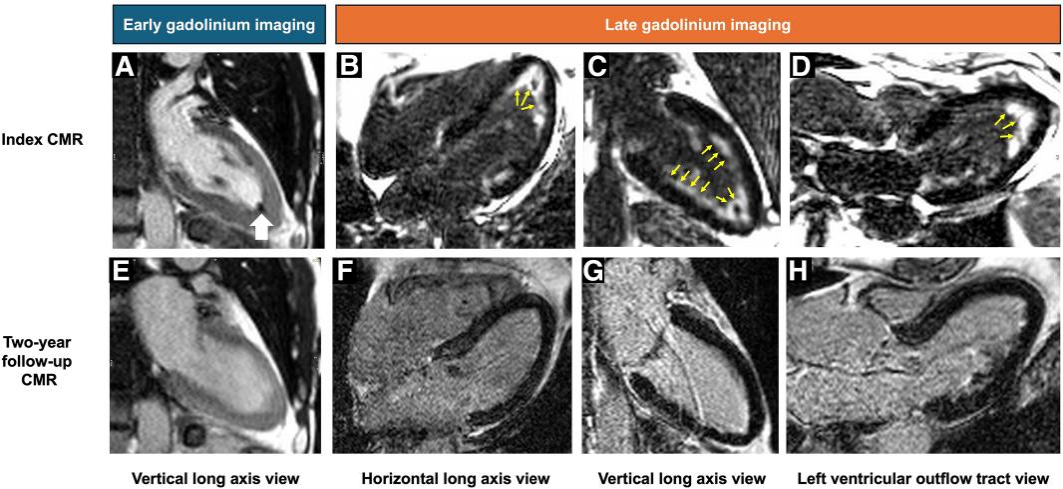


A 69-year-old man presented with dyspnoea, headache, confusion, gait disturbance, paraesthesia, night sweats, and arthralgia. Medical history included sinusitis, nasal polyps, and adult-onset asthma. Blood tests demonstrated eosinophilia (6.8 × 10^9^/L; reference 0.1–0.4 × 10^9^/L), anti-neutrophil cytoplasmic antibodies against myeloperoxidase (>100 U/L; reference 0–9 U/L), and elevated troponin. Electrocardiogram showed lateral T-wave inversion. Cardiac magnetic resonance (CMR) imaging (*Panels A–D*) demonstrated preserved left ventricular (LV) systolic function, apical hypertrophy, diffuse subendocardial late gadolinium enhancement (LGE; yellow arrows), and LV thrombus (white arrow), consistent with eosinophilic myocarditis (EM).

A diagnosis of eosinophilic granulomatous polyangiitis (eGPA) was made, and the patient received warfarin and immunosuppressive therapy: 5-day intravenous methylprednisolone 500 mg, followed by oral prednisolone (60 mg daily, tapered to 20 mg) alongside cyclophosphamide induction (6 g over 6 weeks). Following rapid clinical and biochemical improvement, methotrexate (15mg weekly) was commenced as steroid-sparing maintenance therapy. Two-year repeat CMR showed regression of LGE and resolution of the LV thrombus (*Panels E–H*).

EM is a feature of severe eGPA with three phases: inflammatory, thrombotic, and fibrotic. Pathophysiology involves IL-5 and eotaxin-mediated eosinophil recruitment and activation, followed by degranulation releasing cytotoxic granule proteins that cause cardiomyocyte damage, complement activation, and fibrinoid necrosis. LGE is typically considered a marker of irreversible myocardial damage, but, as this case illustrates, may also represent pharmacologically reversible fibrinoid necrosis changes of inflammatory EM. Reports of improvement with corticosteroid therapy and targeted eosinophil therapy are few. The case highlights the unique value of CMR imaging for diagnosis - and also monitoring of response - in EM.

